# Play-Based Naturalistic Augmentative and Alternative Communication (AAC) Intervention: Educator Fidelity and Effects on Communication and Peer Interactions

**DOI:** 10.3390/bs16081275

**Published:** 2026-07-27

**Authors:** Elizabeth Biggs, Erin Turner, Shana Lee

**Affiliations:** Department of Special Eduation, Vanderbilt University, Nashville, TN 37203, USA; erin.turner.1@vanderbilt.edu (E.T.);

**Keywords:** augmentative and alternative communication (AAC), naturalistic developmental behavioral intervention (NDBI), communication, peer interaction, autism

## Abstract

This study utilized an experimental single-case design to evaluate a professional development package to support educators’ implementation of an augmentative and alternative communication (AAC)-supported, play-based naturalistic developmental behavioral intervention (NDBI), which we called naturalistic AAC intervention (N-AAC). Participants were three minimally speaking students with autism (4–8 years of age), each paired with one of their educators (i.e., speech-language pathologist, pre-service special education teacher, early childhood assistant teacher). The primary aim was to examine the effects of professional development on educator implementation of N-AAC. We also descriptively examined changes in students’ communication and language use during educator–child play and generalization to peer interactions. The findings showed that professional development was effective at teaching educators to implement N-AAC with fidelity. Student communication outcomes showed descriptive improvements following educator training and coaching and N-AAC implementation. However, as expected, gains did not generalize to peer interactions. The findings suggest that professional development improves educator implementation of AAC-supported NDBI strategies during play and that their increased implementation fidelity is accompanied by promising descriptive changes in student communication. Future research is needed to further examine the impact on student communication, promote generalization to peer interactions, and evaluate the potential for larger-scale implementation.

## 1. Introduction

Communication is a fundamental human need and right (Brady et al., 2016), and early childhood is a particularly crucial period for communication development. Learning to communicate provides the foundation for learning and wellbeing throughout a lifespan by enabling children to express their needs, regulate their behavior, learn about themselves and their world, and form relationships. When children lack needed communication support, they are at heightened risk for challenging behaviors, reduced learning opportunities, and social isolation (Iacono et al., 2016). Therefore, educators must support communication development, particularly for children with disabilities who cannot use speech alone to be heard and understood (Beukelman & Light, 2020).

Many children with disabilities have limited speech, including approximately 95% of children with cerebral palsy, 80% with Down syndrome, 50–60% with autism, and 50–60% with other intellectual and developmental disabilities (RERC-AAC, 2019). However, a child’s capabilities to use language extend beyond speech. Augmentative and alternative communication (AAC) refers to ways of communicating that supplement or replace speech, including unaided *AAC* (e.g., gestures, manual signs, nonword vocalizations) and aided AAC (e.g., picture symbols, speech-generating devices [SGDs]) (Beukelman & Light, 2020). Use of aided AAC is an evidence-based practice that can expand children’s vocabulary, support emerging speech, and serve as an enhanced auditory and visual means for language input (Morin et al., 2018; Wood et al., 1998). Aided AAC interventions not only promote children’s language learning and expression but also broader outcomes related to behavior regulation, literacy, and social interaction and peer relationships (M. Romski et al., 2015; Zimmerman, 2025).

High-tech SGDs are especially powerful because they provide access to robust language (i.e., many different words and ways to use grammar to combine words for more complex messages). Educators can promote language learning by using SGDs to model words or ways of combining words within natural interactions. This evidence-based strategy is often called aided language modeling. For example, a teacher might tap the symbol [PAINT] on a student’s SGD while saying “*You are painting!*” during an art activity, helping the student connect spoken words and AAC symbols to their referents and meanings (Biggs et al., 2018; Sennott et al., 2016). SGDs also provide a means of expression, allowing children to communicate about objects, events, and concepts they may not otherwise be able to say (Drager et al., 2010).

Children with autism^1^ represent a substantial portion of those who use or would benefit from aided AAC. Approximately 30–40% of autistic children are minimally speaking or nonspeaking, often defined as using fewer than 30 or 50 spoken words (Koegel et al., 2020; Tager-Flusberg & Kasari, 2013). Autistic children may also benefit from AAC when they use greater than 50 words, as they still may not be able to use speech alone to be heard and understood. This means that approximately half or more of autistic children could benefit from AAC (RERC-AAC, 2019). In a recent survey of educators working with students with intellectual and developmental disabilities, approximately 77% of these special education teachers and 91% of speech-language pathologists (SLPs) reported working with at least one student with autism who used or needed AAC (Biggs et al., 2023). Despite these widespread experiences, educators report needing more support to know how to implement aided AAC and support children’s language and communication learning (Andzik et al., 2019).

Minimally speaking children with autism face distinct challenges that shape the resources and support that educators need to implement AAC-related interventions. One example is joint engagement, which refers to being able to share and sustain attention with another person and an object, event, or activity (Bottema-Beutel et al., 2014). Joint engagement is crucial for language learning and social participation, and so educators need strategies to support children when they have not developed these skills on their own. Another example relates to children’s reliance on prelinguistic communication such as gestures, nonword vocalizations, and facial expressions. These subtle behaviors can be missed by educators, leading to fewer opportunities to reinforce and expand children’s communication attempts (Douglas et al., 2013). Other challenges occur during peer interactions. Peers may ignore or misinterpret non-speech communication, and social exchanges may be brief, overly adult-mediated, or altogether absent (Biggs & Rossi, 2022; Chung et al., 2012). These interconnected challenges illustrate educators’ need for professional development that not only teaches foundational AAC strategies (e.g., aided language modeling) but also how to support children’s engagement in learning activities, so that language models can be more salient.

Naturalistic developmental behavioral interventions (NDBIs) offer a promising solution because they include strategies to support and scaffold children’s joint engagement. NDBIs combine behavioral principles (e.g., prompting, reinforcement, repeated practice) with developmental strategies such as child-led naturalistic intervention and verbal responsiveness (Schreibman et al., 2015). They can be especially effective because they (a) embed teaching within naturally occurring routines, (b) build on children’s interests to promote communication and engagement, and (c) support generalization across settings and adult partners (Schreibman et al., 2015; Tiede & Walton, 2019). Meta-analytic evidence demonstrates that NDBIs are effective at improving communication and language skills for young children with autism, particularly when delivered in natural settings (Crank et al., 2021). However, these outcomes have focused on adult–child communication. Researchers have not evaluated the potential for generalization to peer interactions, but such effects would likely require additional support for peers themselves. Without additional support, peers are not likely to have the same skills and tendencies as adults to act as responsive communication and play partners, particularly with their autistic classmates who have limited speech (Biggs & Rossi, 2022).

NDBIs can focus on spoken language alone, but contemporary evidence indicates they are more effective for children with limited speech when combined with aided AAC (Kasari et al., 2014; Pope et al., 2025). For example, Kasari et al. (2014) conducted a randomized control trial with 61 minimally speaking autistic children ages 5 to 8 years and found that children who participated in an AAC-supported NDBI had better outcomes (i.e., frequency of communication, linguistic diversity, use of communication for social purposes) than children who participated in the same NDBI without AAC. This finding was reinforced in a recent meta-analysis by Pope et al. (2025), which found that AAC-supported NDBIs yielded larger effect sizes on language development for minimally speaking autistic children than speech-only approaches.

Although this evidence points to the promise of AAC-supported NDBIs, significant gaps remain in understanding how to support their implementation in practice, particularly in schools. Nearly all existing research has focused on implementation by familial caregivers or researchers rather than educators (Crank et al., 2021; Pope et al., 2025). Furthermore, descriptive studies suggest educators do not implement AAC or NDBI strategies as effectively as they could (Brady et al., 2010; D’Agostino & Frost, 2025). Therefore, this study evaluated the effects of a professional development package (training and coaching) on educators’ implementation of a play-based, AAC-supported NDBI with preschool and early elementary-aged students. Our research questions (RQs) were as follows: **(RQ1)** Is training and coaching effective for teaching school staff to implement a play-based, AAC-supported NDBI (naturalistic AAC intervention, or N-AAC) with children with autism who are nonspeaking or minimally speaking? **(RQ2)** What is the impact on students’ communication and language use during educator–child play when school staff implement N-AAC? **(RQ3)** Do any gains in communication and language generalize to interactions with peers? **(RQ4)** How do school staff perceive the social validity of N-AAC and the training and coaching provided?

Related to RQ1, we had three aims: (a) to determine whether educators could implement N-AAC with fidelity, (b) to examine changes in educator implementation after in-depth training and coaching, compared to foundational AAC training alone, and (c) to evaluate individual implementation differences across educators. In pursuit of these aims, we implemented foundational AAC training as a preparatory instructional phase through an approach that allowed for comparison of this condition with baseline and the full professional development package. Related to RQ3, we were interested in generalization to interactions with peers because this study was embedded within a larger project focused on improving communication and peer relationships for minimally speaking children with autism. We hypothesized that communication and language gains during adult-mediated intervention would not generalize to peer interactions, making it important to explicitly support peers as communication partners (e.g., Biggs et al., 2025). However, this specific hypothesis was important to test because it had not previously been empirically tested by our research team or others.

## 2. Methods

### 2.1. Participants

Participants were three dyads, each with one educator and one autistic student who was nonspeaking or minimally speaking. To be included, focus students needed to: (a) be enrolled in a public or private school in grades preK–3rd grade, (b) receive special education services as a student with autism, and (c) be nonspeaking or minimally speaking, defined as using fewer than 30 spoken words based on observation and educator report. To recruit, an email was sent to educators from an urban, public school district and an independent inclusive preschool in the same metropolitan area. Four educators responded, each from a different school. Parent consent was obtained for one student at each school; all were screened and found eligible. However, we could not implement this study with one student because he began having absences due to a medical condition prior to beginning baseline. Therefore, this study reports on three students, each paired with an educator from their team who was interested in receiving training and coaching. The educators held a range of professional roles and included a pre-service special education teacher, an SLP, and an early childhood assistant teacher.

### 2.2. Emmet and Abby, a Pre-Service Special Education Teacher

Emmet (all names are pseudonyms) was a 6-year-old (kindergarten) Black male with autism (age of diagnosis unreported). English was his primary language. He was an emerging symbolic communicator who primarily communicated to request and reject through gestures and nonword vocalizations. Emmet did not spontaneously use spoken words, but he sometimes verbally imitated words when prompted (e.g., “All done” “help”). He did not have access to aided AAC at the start of this study. Receptively, Emmet understood most single-step directions when paired with gestures. A research team member administered the Peabody Picture Vocabulary Test, Fifth Edition (PPVT-5) to assess receptive vocabulary, and Emmet received a standard score of 40 (percentile rank < 0.1%). We also conducted a 15 min play-based, structured language sample with a research team member to gather additional information about Emmet’s communication skills. Emmet communicated intentionally seven times in the language sample, each time to request or reject using gestures or body movements. He did not imitate or spontaneously produce any words during the language sample.

Abby was a 23-year-old White female and a pre-service teacher working towards her master’s degree in special education and applied behavior analysis. Abby reported she had received AAC training through formal coursework and by reading research articles and textbooks. She had three years of supervised field experience working with students with disabilities during her undergraduate and master’s programs.

### 2.3. Leander and Rachel, an SLP

Leander was an 8-year-old Black male (3rd grade), diagnosed with autism at age three. English was his primary language. Leander was an emerging symbolic communicator who primarily communicated to request and reject through pointing, body movements (e.g., taking people’s hands to something), and nonword vocalizations. He produced no spoken words but was beginning to use aided AAC (i.e., an iPad with TD Snap Motor Plan) (Apple, Cupertino, CA, USA; Tobii Dynavox, Coraopolis, PA, USA). This AAC device was provided by the school, and Leander had been using it for just over a month when this study began. His team reported that he used AAC primarily during speech therapy, specifically when prompted or asked a direct question. Receptively, Leander understood some single-step directions with gestures, and he received a standard score of 40 on the PPVT-5 (percentile rank < 0.1%). During the 15 min language sample, Leander engaged in vocal stimming but did not use words or nonword vocalization to communicate intentionally. He communicated four times, each time using body movements or gestures to request. Leander’s AAC device was present during the language sample, but he did not use it to communicate.

Rachel, a 37-year-old White female, had 12 years of experience as an SLP and 9 at Leander’s school. Rachel reported participating in several types of AAC-related professional development in the last five years, including workshops, webinars, a professional learning community, and reading peer-reviewed research articles. However, she still described AAC as being one of the most difficult parts of her job.

### 2.4. Hallie and Megan, an Early Childhood Assistant Special Education Teacher

Hallie was a 4-year-old Black female with autism (age of diagnosis unreported) whose primary language was English. She attended PreK at a private inclusive preschool. Hallie was an emerging symbolic communicator. She primarily used gestures and nonword vocalizations but also produced approximately 15 spoken words. Hallie had been using AAC for about a year (i.e., an iPad with the Core First vocabulary set on TD Snap, obtained through insurance) (Tobii Dynavox). She used her AAC device at school when teachers prompted her but not on her own. She took the device home, but her parents reported she rarely used it. Receptively, Hallie sometimes followed single-step directions when paired with visuals or gestures. She received a standard score of 40 (percentile rank < 0.1%) on the PPVT. In the 15 min language sample, Hallie communicated intentionally 11 times, mostly using gestures and nonword vocalizations to request objects or help. She imitated three spoken words (i.e., “uhoh”, “falling”, “make”) but did not produce any spontaneous words with speech or AAC.

Megan, a 23-year-old White woman, was the early childhood assistant teacher in Hallie’s PreK classroom. This was Megan’s first year in a classroom role. She reported that she had participated in a school workshop about AAC and received informal support related to AAC from other school staff she worked with.

### 2.5. Peer Participants

Six peers without disabilities also participated, two from each student’s school and grade. We asked educators to identify peers who (a) liked spending time or talking with the focus student, (b) had age-appropriate social and communication skills, and (c) had good school attendance. Emmet’s peers were a Black girl and a Black boy. Leander’s peers were a White girl and a White boy. Hallie’s peers were a White girl and a boy (race/ethnicity not reported).

### 2.6. Setting

This study took place in the students’ schools. Leander and Emmet attended different elementary schools in the same large, urban public district. Each school served approximately 400 students in kindergarten to 5th grade. Leander’s school was about 53% White, 31% Black/African American, 9% Hispanic/Latino, and 7% multiracial; approximately half of students qualified for free or reduced-price lunch based on income. Emmet’s school was about 17% White, 77% Black/African American, 2% Hispanic/Latino, and 4% multiracial; nearly 90% qualified for free or reduced-price lunch. Hallie attended a private inclusive preschool in the same urban area serving about 90 students, one-third of whom had disabilities. Her school was about 70% White, 18% Black/African American, 11% Asian, and 1% Hispanic/Latino.

### 2.7. Experimental Research Design and Procedures

The institutional review board (Vanderbilt University, #230692) approved this study prior to recruitment. All participants provided informed consent/assent. Given the age and developmental levels of students with autism and peers, we assessed signs of behavioral assent or dissent for each session, starting with a question like “Want to come and play?” Focus students and peers showed only assenting and no dissenting behaviors for all sessions. Sessions would have been stopped or not held if dissenting behaviors were observed.

This study used a nonconcurrent multiple-baseline-across-participants design (Slocum et al., 2022). Scheduling and resource constraints precluded a concurrent design, but this study met contemporary guidelines for a nonconcurrent design being an appropriate choice (Ledford & Zimmerman, 2023; Slocum et al., 2022). The intervention was introduced systematically across dyads using randomized baseline lengths (3, 6, or 9 sessions). Educator N-AAC fidelity was the primary dependent variable (DV), and so data collection stopped when an educator reached a pre-determined threshold, defined as 70% fidelity for three consecutive sessions without coaching (see *Educator Implementation of N-AAC Strategies*). Planned maintenance probes were unable to be conducted due to the end of the school year for participants. Following single-case guidelines, data were analyzed using visual analysis of the level, trend, variability, consistency, overlap, and immediacy of effect (i.e., within and between conditions, across educator–child dyads) (Ledford et al., 2018).

### 2.8. Pre-Baseline Procedures

Prior to baseline, we collaborated with school teams to: (a) identify play materials, (b) establish AAC access, and (c) schedule sessions.

**Play Materials.** We assessed student play preferences through observation and educator interviews and then encouraged educators to rotate toys from this list, so long as they incorporated student interests and always had two toy sets available that represented different types of play (i.e., exploratory, construction, and functional/pretend play). Emmet often played with building toys (e.g., magnet tiles, marble run) and playdoh with tools. Leander often played with animal figurines and exploratory toys (e.g., sand, fidgets, putty). Hallie often played with blocks and pretend-play toys (e.g., animals, birthday cake or tea set, baby dolls).

**AAC Access.** Leander and Hallie used their own iPad AAC devices: Leander with TD Snap Motor Plan (6 × 5 grid) and Hallie with TD Snap Core First (6 × 5 grid). Emmet did not have an AAC device, so we collaborated with the educational team to determine an appropriate device. Through feature matching, we chose to trial TouchChat (PRC-Saltillo, Wooster, OH, USA) and TD Snap with the Motor Plan page set (both as apps on an iPad). During a 20 min play-based trial, a research team member introduced each AAC system and modeled its use, encouraging Emmet to also explore and use the apps. Following the trial, we selected the iPad with TD Snap with the Motor Plan page set (6 × 5 grid) because Emmet attended to it more during the trial (as shown by visual gaze and imitating the pressing of symbols when directly encouraged to do so). All the AAC devices had robust language systems with diverse vocabulary (i.e., nouns, verbs, descriptors, high-frequency functional words) and features such as search functions and masking capabilities (i.e., temporarily hiding icons) to support navigation. Together with the participating educator, we identified 20–25 target words (“teaching” words) that the student did not already produce with speech or AAC. Target words had to include nouns (e.g., baby, sand), verbs (e.g., make, eat), descriptors (e.g., fun, fast), and high-frequency functional words (e.g., go, more, want). Devices remained programmed with full vocabulary, but we taught educators how they could temporarily mask non-target words.

**Scheduling.** Sessions were intentionally brief so that they could be scheduled naturally within the school day. We asked educators to hold sessions 2–3 times per week but allowed them to determine the schedule and location. Rachel held sessions in her shared SLP office space. Abby and Megan decided to hold sessions in rooms outside the classroom to reduce distractions, which included the library, a conference room, and rooms designed for 1:1 therapy or intervention. All students continued to receive their regularly scheduled special education services and instruction throughout baseline and intervention.

### 2.9. Baseline

Baseline consisted of 10 min 1:1 play sessions with the educator and student. Educators were directed to play naturally and try to support children’s language learning. We asked them to (a) provide two toy sets across types (i.e., exploratory, construction, pretend/functional play), based on student interest, (b) sit and play with the student, (c) make sure the AAC device was available, and (d) clean up with the student when the timer went off.

### 2.10. Intervention

The intervention consisted of training and coaching for educators to implement a play-based, AAC-supported NDBI called N-AAC. Prior to this study, we spent about nine months developing N-AAC through a community-engaged approach with students, educators, and families at Hallie’s preschool. The pilot first involved researchers conducting N-AAC sessions and later involved training and coaching two educators to implement N-AAC sessions (each with one student). Through this work we (a) defined intervention strategies, (b) developed training and coaching materials with video models, and (c) developed and refined a fidelity rubric, all while incorporating educator feedback. Hallie and Megan were not involved in the pilot and therefore had no exposure to the intervention prior to this study.

**Figure 1:**
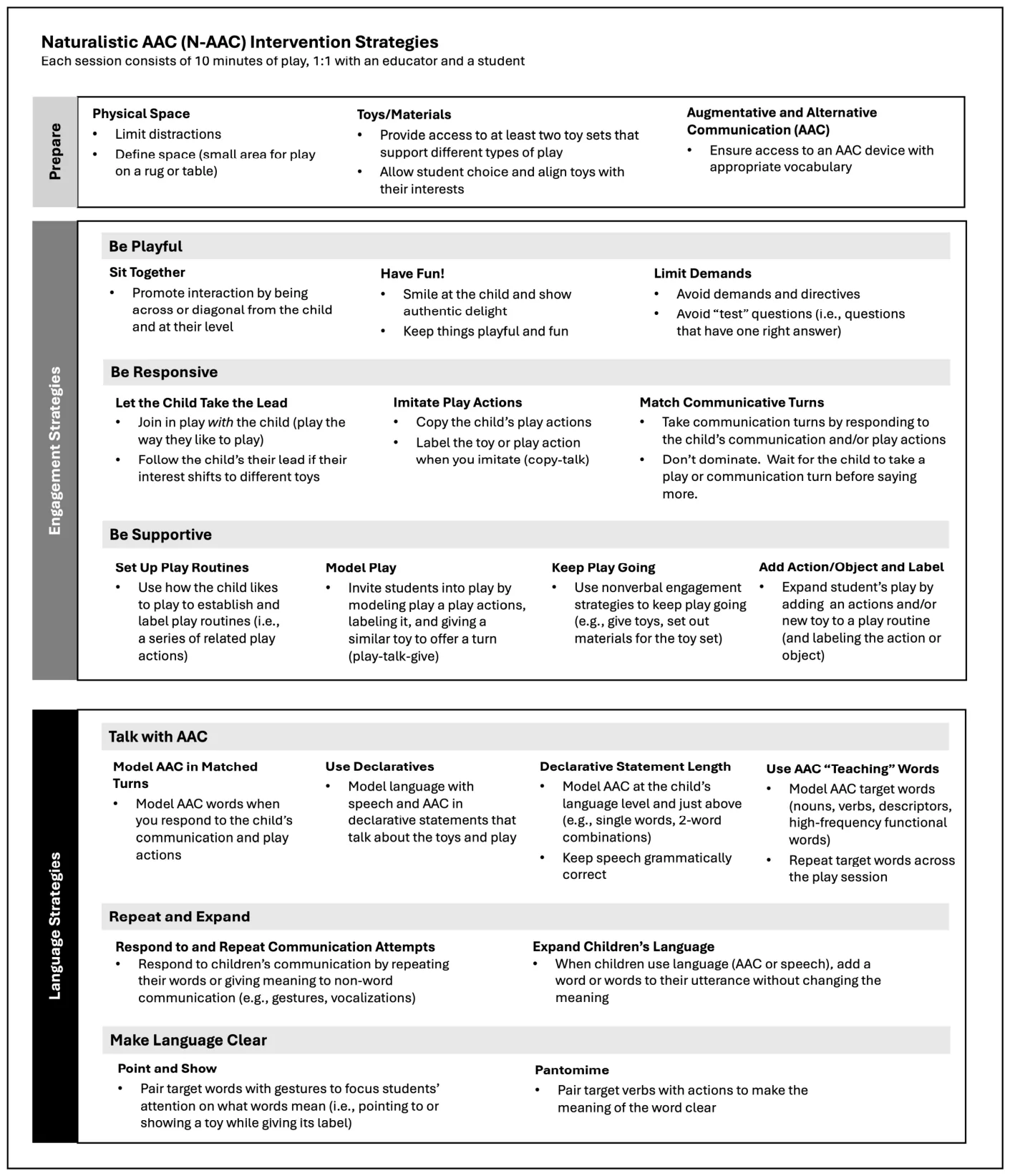
An Overview of Naturalistic AAC (N-AAC) Intervention Strategies.

**Naturalistic AAC Intervention (N-AAC).** N-AAC consisted of AAC-supported NDBI strategies, which focused on scaffolding joint engagement in play and then modeling and teaching language within this supported engagement. Strategies included setting up the environment, joining in and supporting play, responding to and expanding child communication, and modeling language with speech and AAC. There were three N-AAC components (shown in Figure 1): Prepare, Engagement strategies, and Language strategies. Prepare focused on the physical space by limiting distractions and ensuring access to toys and the AAC device. The Engagement and Language components were divided into “toolkits”, each with 2–4 intervention strategies. The Engagement toolkits were: (a) Be Playful (i.e., sit together, have fun, limit demands), (b) Be Responsive (i.e., let the child take the lead, imitate play actions, match communicative turns), and (c) Be Supportive (i.e., set up play routines, model play, keep play going through nonverbal engagement strategies, expand play by adding an object or action with a label). The Language toolkits were: (a) Talk with AAC (i.e., model AAC in matched turns, use declarative statements, model language at or just above the child’s level, use AAC “teaching” word), (b) Repeat and Expand (i.e., respond to and repeat communication attempts, expand children’s language), and (c) Make Language Clear (i.e., point and show, pantomime). Figure 1 provides additional information about the N-AAC intervention strategies.

**Training and Coaching.** The professional development package consisted of three components: (1) initial training on AAC fundamentals followed by independent practice sessions, (2) in-depth N-AAC training and practice-based coaching, and (3) ongoing coaching (that included self-reflection, feedback, problem solving, and setting goals). The intervention coach provided all training and coaching. She was a member of the research team who was an experienced special education teacher and instructional coach, and she had experience implementing N-AAC during the pilot.

***Initial Training on AAC Fundamentals.*** This first component included (1) a 1 h didactic training focused on foundational information about AAC-related intervention and (2) three independent practice sessions with the student. A key focus of the training was on teaching aided language modeling, which consists of modeling AAC within natural interactions (e.g., saying, “stack the block” while pressing [STACK] and modeling the play action) (Biggs et al., 2018). Although this training provided foundational knowledge about AAC and aided language modeling, it did not include other information about N-AAC strategies. The coach delivered training individually to an educator either on Zoom or in person (educator preference) using a PowerPoint with visuals and explanations, video models, discussion and reflection questions, and time for questions and planning. Training covered five topics: (1) welcome, (2) introduction to AAC, including myths and misconceptions, (3) key principles (e.g., selecting target words, device navigation), (4) aided language modeling, and (5) wrap-up and reflection. After the training, each educator held three independent play-based practice sessions with the student to familiarize themselves with the student’s AAC device and aided language modeling. During the AAC training phase, educators did not receive additional coaching or support beyond the 1 h training. This component lasted 1–2 weeks, depending on educators’ schedules.

***N-AAC Training and Practice-Based Coaching.*** The second component consisted of (1) two 1 h didactic training sessions and (2) two 20 min practice-based coaching sessions. Didactic training sessions used the same format as the initial training but focused specifically on N-AAC intervention strategies (see Figure 1). The first covered Engagement strategies (including Prepare) and the second covered Language strategies. Practice-based coaching sessions occurred in person with the student, educator, and coach. One practice-based coaching session was held after the engagement training and one followed the language training, allowing educators to practice strategies before learning new content. During each, the coach: (1) collaborated with the educator to set a goal to focus on, (2) provided real-time coaching (e.g., modeling, sharing information, feedback), (3) faded support to build educator independence, (4) addressed educator questions, and (5) concluded with a shared summary and reflection. It took approximately two weeks to complete both training sessions and practice-based coaching sessions (1 week each for engagement and language).

***Ongoing Coaching.*** Following N-AAC instruction, post-session coaching was provided weekly for the first two weeks. Subsequent coaching occurred if two consecutive sessions fell below 70% fidelity or if the educator requested it. Each educator received one additional coaching session beyond the planned two. Coaching lasted 10–15 min and occurred 1–2 days after a student session, either in person or via Zoom (educator preference). Prior to coaching, the coach reviewed the most recent video-recorded sessions and asked the educator to reflect on what went well and what was challenging. During coaching, the coach: (1) facilitated educator reflection sharing, (2) collaboratively addressed challenges, (3) provided specific feedback (positive and constructive), and (4) helped the educator set a goal. Coaching strategies included problem solving, role playing, video examples, and discussing fidelity data.

### 2.11. Implementation Fidelity

Implementation fidelity was scored for all training and coaching sessions. A trained research assistant evaluated the coach’s adherence using checklists, while reviewing video or audiotaped training and coaching sessions. Implementation fidelity for training sessions was as follows: AAC fundamentals (16 steps, 100% fidelity), N-AAC engagement (28 steps, 98.8% fidelity), and N-AAC language (26 steps, 97.4% fidelity). The steps missed were minor (e.g., omitting a prompt to review or recall information). Implementation fidelity for all practice-based coaching (6 steps) and post-session coaching sessions (8 steps) was 100%. Detailed fidelity checklists are provided on Open Science Framework (OSF): https://osf.io/9ba3c/overview?view_only=1d11c2191dd342fba4a7c169c7fe2394 (accessed on 1 June 2026).

### 2.12. Dependent Variables (DVs) and Observational Data Collection

Educators self-recorded baseline and intervention sessions using a loaned camera (GoPro8 or GoPro10). Sessions lasted approximately 10 min but ranged from 9 to 14 min due to the natural context. For analysis, videos were standardized to 8 min clips by cutting the first 60 s and retaining the next 8 min.

### 2.13. Transcription of Educator–Child Play Sessions

Each session was transcribed by trained research assistants using guidelines adapted from the Systematic Analysis of Language Transcripts (SALT; Miller et al., 2019). SALT guidelines were adapted for use within Datavyu version 1.5.3 (https://datavyu.org), an open-source software for observational coding. This approach enabled time stamping of utterances and integrated video playback, supporting accurate transcription of subtle prelinguistic communication (e.g., gestures, vocalizations). Research assistants transcribed educator utterances (speech and/or AAC) and child intentional communication using one Datavyu cell per C-unit. A C-unit comprises a main clause or a main clause with subordinating clauses (Miller et al., 2019). Children’s linguistic and prelinguistic communication acts were transcribed, so long as they showed evidence of being intentional communication to the educator. Evidence of communicative intent required (a) use of contextually relevant language (speech/AAC), (b) orienting to the adult (e.g., eye contact, head turn, touch), *or* (c) responding contingently to adult communication. Behaviors without evidence of communicative intent were not transcribed, with one exception: potential communicative behaviors (PCBs) were defined as children’s nonword vocalizations or exploratory AAC use without clear intent but that could be assigned meaning by the educator. To support coding of educator verbal responsiveness as an N-AAC strategy, PCBs were transcribed when educators responded to them. However, PCBs were transcribed in a different Datavyu code prompt than intentional communication to distinguish them from one another. Only intentional communication was included in calculations for analysis. To ensure accuracy, all transcripts were verified by a second trained research assistant. The research team met weekly to resolve transcription questions and disagreements collaboratively (Yoder et al., 2018). The full transcription manual is available on OSF (see previous hyperlink).

### 2.14. Educator Implementation of N-AAC Strategies (Primary DV)

**Figure 2:**
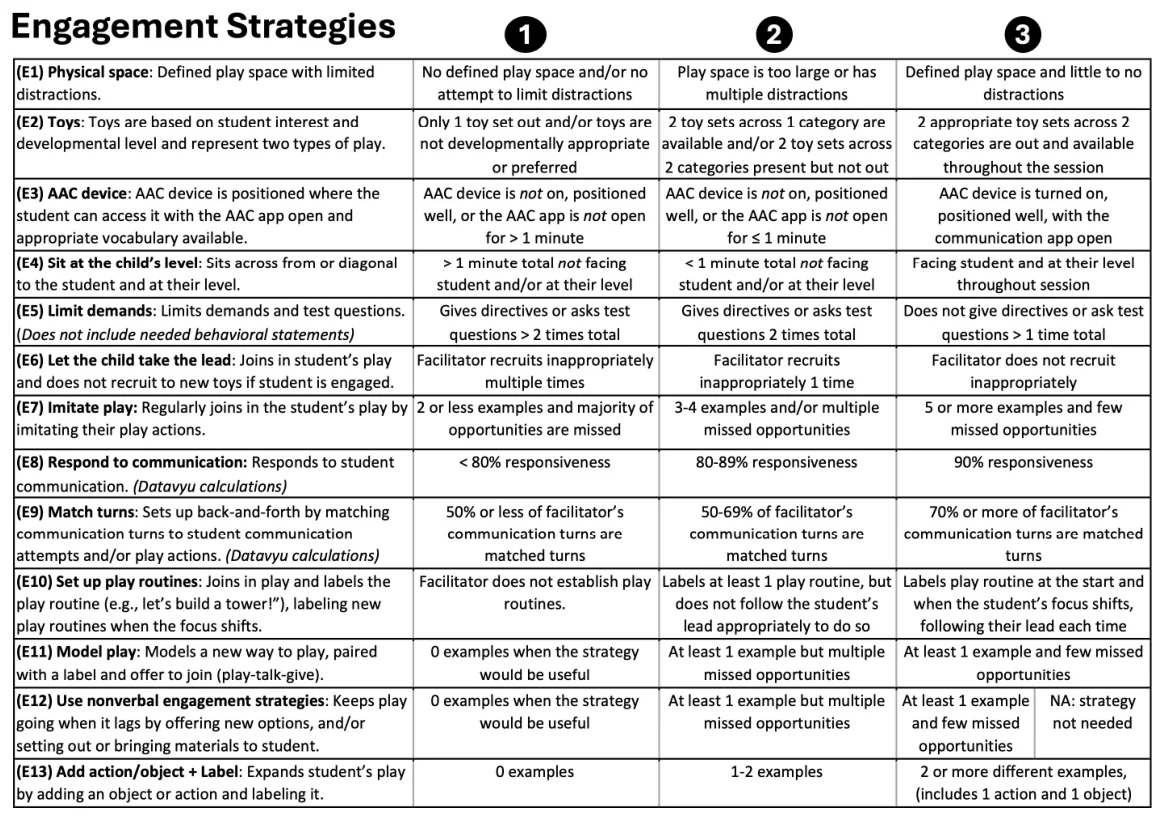
N-AAC Fidelity Rubric Items for Engagement Strategies.

**Figure 3:**
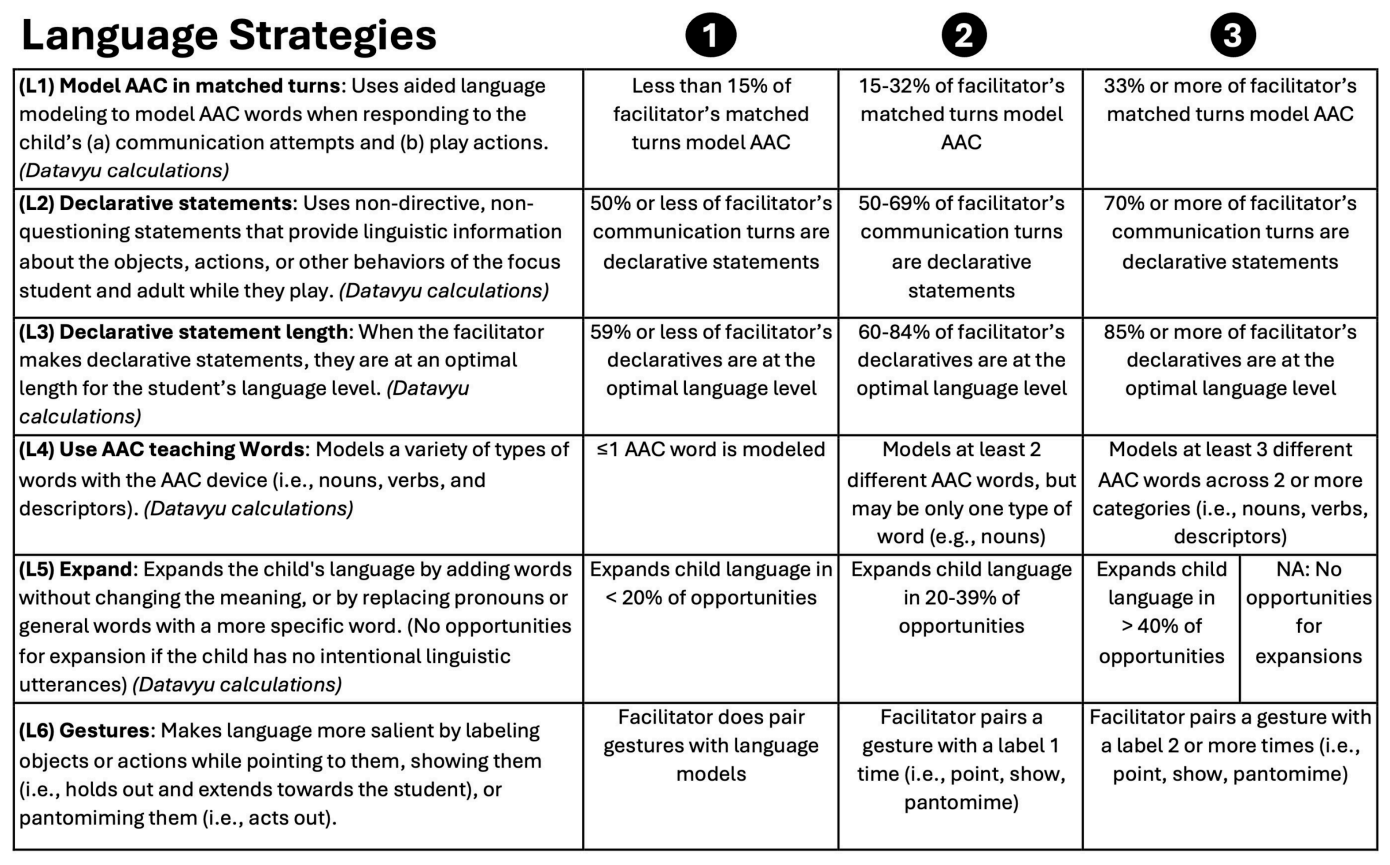
N-AAC Fidelity Rubric Items for Language Strategies.

The primary DV was educators’ implementation of N-AAC strategies, measured as a percent (out of 100%) using an observational fidelity rubric. The rubric was stringent so it would be sensitive for measuring growth (i.e., required very strong implementation to achieve a high percent fidelity), and so benchmarks accordingly were: <70% *not implemented with fidelity*, 70–85% *implemented with fidelity*, and >85% *implemented with excellent fidelity*. The rubric and benchmarks were developed collaboratively with educators at the inclusive preschool during the pilot (see Section 1). This extensive process involved repeatedly scoring researcher and educator fidelity during the pilot and then using observational insights and educator feedback to (a) make incremental adjustments to individual criteria on the rubric and (b) determine the 70% benchmark for fidelity. The rubric has 19 items, 13 related to Engagement (E) and 6 to Language (L): physical space (item E1), toys (E2), AAC device (E3), sit at the child’s level (E4), limit demands (E5), let the child take the lead (E6), imitate play actions (E7), respond to communication attempts (E8), match turns (E9), set up play routines (E10), model play (E11), use nonverbal engagement strategies (E12), add an object/action + label (E13), model AAC in matched turns (L1), use declarative statements (L2), declarative statement length (L3), use AAC teaching words (L4), expand language (L5), and use gestures to make language models salient (L6). Figure 2 (Engagement) and Figure 3 (Language) overview the items, and the full rubric is available on OSF (see previous hyperlink).

Each item was weighted equally and scored 0, 1, or 2 based on operationalized definitions (see Figure 2 and Figure 3). Twelve items (11 Engagement, 1 Language) were scored through global ratings, in which a trained research assistant viewed the 8 min video and assigned a score for each item based on operational definitions, viewing the video as many times as needed. The remaining seven items (2 Engagement, 5 Language) required discrete behavioral counts. For example, respond to communication attempts (E8) calculated the percent of times that educators responded (with speech or AAC) to child communication. To score these items, trained research assistants first used Datavyu to apply five codes to each educator utterance on the transcript: (1) AAC use (1 = yes, 0 = no), (b) utterance type (e.g., declarative, question, directive), (c) matched turn (1 = turn-taking response that was contingent on child communication or play behavior; 0 = no), (d) if applicable, matched turn type (i.e., linguistic expansion, other response to child communication, response to child play behavior), and (e) declarative length (for declarative statements only). Then, the research assistant used Excel templates to export Datavyu codes, run appropriate calculations, and then assign the relevant rubric score. Fidelity percentages were then calculated by dividing the total score (i.e., sum of 0–2 scores for all items) by the total possible score of 38 and then multiplying by 100%. In addition to calculating a percent of total fidelity, we also calculated (and graphed) separate fidelity scores for engagement and language strategies to monitor implementation across these areas. Complete Datavyu coding procedures are available on OSF (see previous hyperlink).

### 2.15. Focus Student Intentional Communication and Language Use (Secondary DV)

Secondary DVs included focus student intentional communication and language use during play sessions with educators. Research assistants coded each transcribed child utterance for form and function. Form comprised two inter-related categories of intent and mode. *Intent* codes were: (1) intentional linguistic (i.e., purposeful words with speech and/or AAC), (2) intentional prelinguistic (i.e., nonword vocalizations, unintelligible speech, and/or gestures accompanied with evidence of communicative intent), (3) non-discriminatory AAC use (i.e., pressing icons *with* evidence of communicative intent but *without* appropriate discrimination, such as pressing [HIPPO] instead of [MONKEY]), and (4) PCB (i.e., nonword vocalizations or AAC exploration without communicative intent but to which the educator responds). *Mode* was coded only for intentional communication (not PCBs) and marked the presence or absence of gestures/body movements, nonword vocalizations/unintelligible speech, aided AAC, and intelligible speech. *Function* (communicative purpose) was coded only for intentional communication involving language or gestures, not for nonword vocalizations or non-discriminatory AAC use (for which function is difficult to identify with accuracy) or PCBs (which are not intentional communication). Function codes were: (1) joint attention (JA), (2) behavior regulation (i.e., request, protest) (BR), (3) imitated or gesture-only response (i.e., child response to an adult’s declarative or open-ended question that did not involve the child producing spontaneous language with speech or AAC) (RES-I), (4) spontaneous linguistic response (i.e., a child’s response to an adult’s declarative or open-ended question that involved the child producing spontaneous language) (RES-S), and (5) a response to a prompt or “test” question (i.e., child communicative response to a prompt or a question with a single answer like “what color is this?”) (RQP). Complete Datavyu coding procedures are available on OSF (see previous hyperlink).

Datavyu codes were used to calculate and graph two main variables: (a) unprompted intentional communication (i.e., prelinguistic and linguistic intentional communication, excluding RQP) and (b) unprompted linguistic communication (i.e., unprompted intentional communication that involved purposeful spoken or AAC words). Research assistants also calculated the number of cumulative new words (Rogers-Warren & Warren, 1980) to track vocabulary growth. A word was counted toward the number of cumulative new words if it was (a) produced with speech or AAC, (b) within an unprompted intentional linguistic communication act, and (c) was not produced in any previous session. Novel words were recorded for each session and added to the cumulative total. Words counted only once regardless of mode (e.g., speech, AAC, both). For instance, in Session 5 Hallie said [HELP] with AAC and the letter name “S” with speech (cumulative count: 2). Then, in Session 6 she said [HELP] again and “ta-da!” with speech (cumulative count: 3).

### 2.16. Communicative Interactions with Peers During Play Probes (Secondary DV)

Secondary DVs also included communicative interactions with peers during play probes, which were conducted across baseline and intervention conditions. We intended to conduct one peer play probe for every 3–4 adult–child sessions, but student absences altered this slightly (five total for Emmet and Hallie, four for Leander). A structured protocol was used for each 5 min play probe, which was videotaped. A research assistant presented 2–3 toy sets representing at least two types of play (exploratory, construction, pretend/functional) that were commonly used during adult play sessions. The research assistant had all three students sit in a defined play space so that students were in physical proximity to each other (i.e., at a table or on a rug on the floor), ensured the AAC device was on and in reach of the students, introduced the activity, and then moved away to avoid intervening unless children (a) tried to leave the play area, (b) engaged in unsafe behavior, or (c) were not playing for >1 min (in which case they offered a suggestion or new toy). Play probes were transcribed and coded in Datavyu using simplified procedures from the adult–child sessions. Specifically, codes were assigned for: (1) focus student intentional communication to peers (i.e., prelinguistic or linguistic communication directed to peers) and (2) peer communicative interactions with the focus student (i.e., spoken words or AAC use directed to the focus student).

### 2.17. Observer Training and Inter-Observer Agreement (IOA)

Prior to coding, research team members (seven student research assistants and one research staff member) were trained to reliability standards on one or more of the following: (a) adult–child Datavyu coding, (b) fidelity rubric scoring, or (c) peer play probe Datavyu coding. For each role, training included reviewing coding manuals, 3–5 h of instruction, practice coding, and passing two test videos with ≥85% point-by-point agreement.

Inter-observer agreement (IOA) was calculated for all coding types. Twelve adult–child sessions (21.1%) were randomly selected for a second coder, balanced across participants and conditions. For Datavyu coding, IOA averaged 91.4% for adult variables and 97.2% for focus student variables. Specifically, average IOA was 98.9% for adult use of AAC (95.8–100.0% across sessions), 88.0% for adult utterance type (77.6–100.0%), 86.2% for adult matched turns (76.8–92.5%), 86.0% for adult type of matched turn (76.8–94.3%), 97.7% for adult declarative statement length (91.4–100.0%), 99.5% for focus student mode of communication (96.0–100.0%), and 83.0% for focus student function (60.0–100.0%). For fidelity rubric scoring, IOA averaged 93.0%. Several items were scored at 100% accuracy: physical space (item E1), toys (E2), AAC device (E3), respond to communication attempts (E8), match turns (E9), model AAC in matched turns (L1), use declarative statements (L2), and declarative statement length (L3). IOA ranged from 75.0% to 91.7% for all other rubric items, with the lowest IOA for limit demands (E5) (75.0%). IOA for all other rubric items was over 80%. Finally, 6 of the 14 peer play probe sessions were randomly selected for IOA coding (42.9%), balanced across participants. Average IOA was 100% for peer communicative interactions to the focus student and 83.3% for focus student intentional communication to peers.

### 2.18. Social Validity

Social validity was assessed on goals, procedures, and outcomes using an electronic REDCap questionnaire (Harris et al., 2009) that consisted of open-ended questions and ratings. We invited the three participating educators to complete the social validity questionnaire after this study, along with one other educator from each student’s team. Leander’s special education teacher declined to participate, but Emmet’s paraeducator and a pre-service teacher working in Hallie’s classroom both participated. For educators who completed the training and coaching, the social validity questionnaire took 15–20 min and included: (1) open-ended questions about the goals, procedures, and outcomes, (2) ratings of comfort with each N-AAC strategy (5-point scale, from 1 = *very uncomfortable* to 5 = *very comfortable*, with an option to instead respond “*I don’t remember this strategy*”), (3) ratings about the usefulness of each N-AAC strategy (5-point scale, from 1 = *not at all useful* to 5 = *very useful*, with the option “*I don’t remember this strategy*”), and (4) quality ratings of four video clips (two baseline, two intervention). For the video ratings, 1 min clips were randomly selected from baseline and the final three N-AAC sessions. They were presented in a random order without labels of being from baseline or intervention (to avoid influencing educators’ ratings). Educators rated each clip on six items (i.e., student quality of engagement with the adult, engagement with the toys, communication and educator quality of engagement support strategies, communication support strategies, responsiveness). Ratings used a 5-point scale from 1 = *very low* to 5 = *very strong*. The other two educators completed a briefer questionnaire (10 min) with: (1) open-ended questions about study goals and outcomes and (2) the quality ratings of the same four video clips.

## 3. Results

### 3.1. RQ1: Effectiveness of Training and Coaching on Educator Implementation

**Figure 4:**
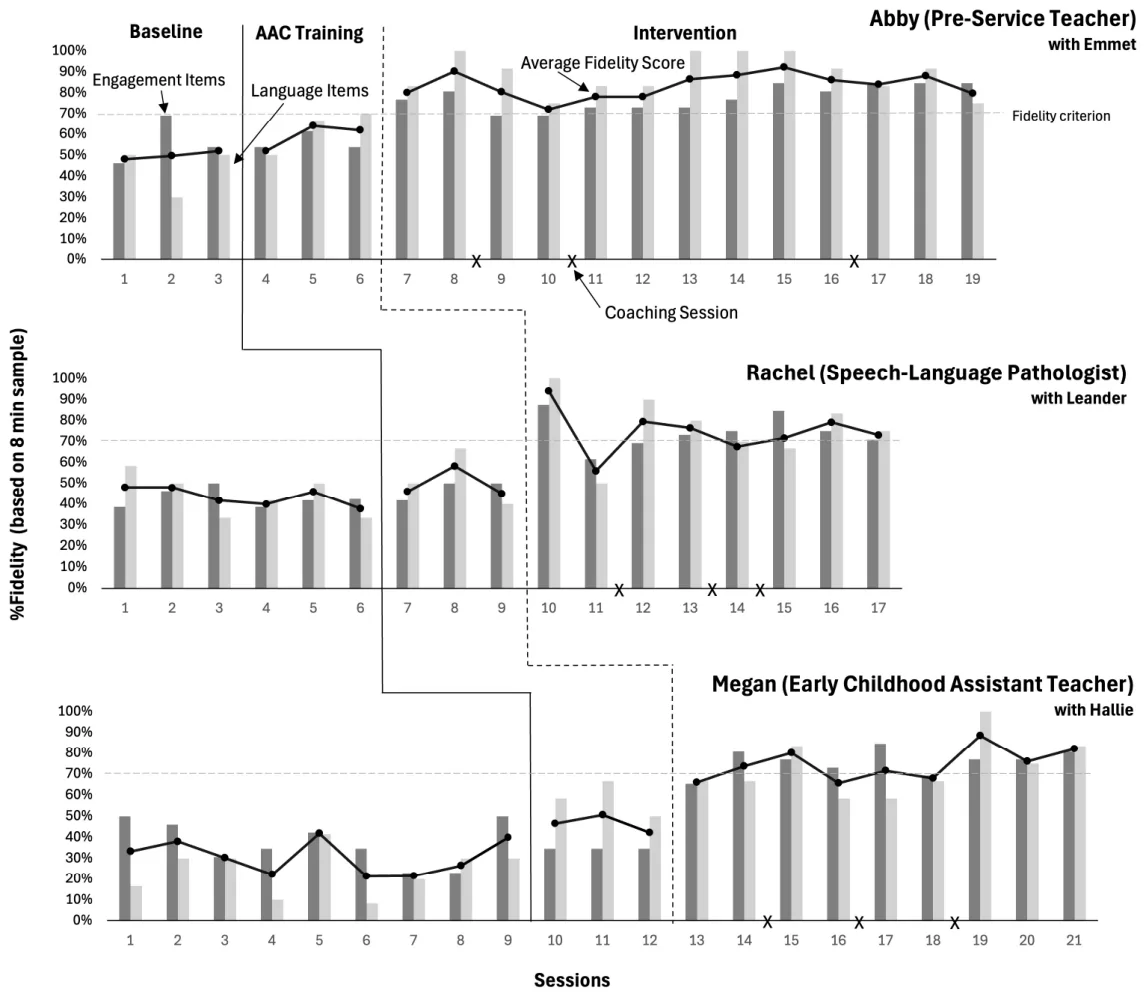
Primary Single-Case Graph Depicting the Impact of Professional Development on Educators’ Implementation of N-AAC.

**Figure 5:**
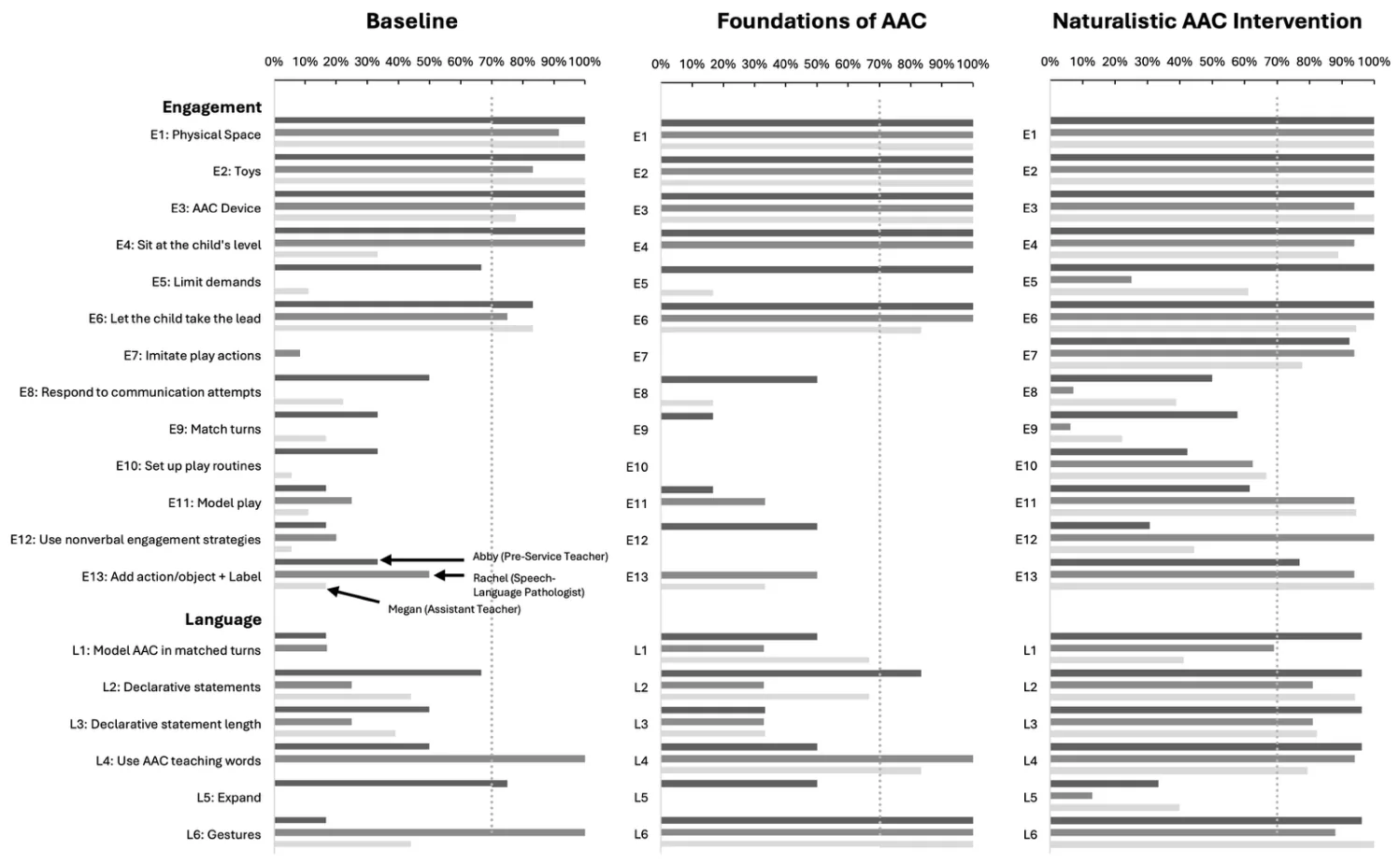
Descriptive Findings about Educators’ Average Use of each N-AAC Strategy across Study Conditions.

Figure 4 displays educator N-AAC implementation across baseline and intervention sessions. Professional development was effective, evidenced by the functional relation between the training and coaching and educator fidelity. Visual analysis showed low to moderate implementation in baseline for each educator (*M* = 41.4%; range, 21.5–51.9% across educators and sessions). Educators reached the criterion for implementation (i.e., 70% fidelity for a minimum of three consecutive sessions without additional coaching) only after receiving the N-AAC training and practice-based coaching, not after the initial AAC training. Individual differences were observed in educators’ (a) baseline strategy use, (b) growth trajectory, (c) fidelity level at the end of this study, and (d) strengths and challenges with specific strategies. Figure 5 visually depicts information about educators’ use of each N-AAC strategy across conditions, and individual differences are described below.

Note, solid lines on Figure 5 represent the primary dependent variable of percent fidelity for N-AAC implementation. Criterion was set at 70% fidelity for three consecutive sessions without coaching. Darker gray bars show the percent fidelity for engagement items within the N-AAC rubric, and light gray bars show the percent fidelity for language items. An “X” marks post-session coaching.

### 3.2. Abby (Pre-Service Teacher)

Average baseline fidelity for Abby was 49.9%, with stronger Engagement than Language strategy use. Abby demonstrated strengths in Prepare strategies (physical space, toys, AAC device), sitting at the child’s level, and letting the child take the lead. She modeled AAC occasionally (*M* = 3.6 times/baseline session) but rarely in matched turns. She rarely used play strategies like setting up play routines, imitating play actions, or modeling or expanding play. Abby’s AAC modeling increased slightly after initial training (*M* = 5.3 times/session), with no other meaningful changes. She achieved 70% fidelity immediately after N-AAC training and practice-based coaching, maintaining fidelity (70–85%) or excellent fidelity (>85%) levels for all intervention sessions (46% at excellent fidelity). Fidelity averaged 83.5% during the full intervention condition, with the greatest growth in using language strategies (AAC modeling in matched turns, using AAC teaching words, declarative statements, and using gestures paired with language models). She modeled AAC frequently after N-AAC instruction (*M* = 25.5 times/session). Some engagement strategies remained challenging for Abby (e.g., setting up play routines, modeling play, nonverbal engagement strategies), but they still improved meaningfully from baseline.

### 3.3. Rachel (Speech-Language Pathologist)

Average baseline fidelity for Rachel was 43.6%, with variable use of Engagement and Language strategies. Rachel showed strengths in Prepare strategies (physical space, toys, AAC device), sitting at the child’s level, using AAC teaching words, and pairing gestures with language models. She frequently modeled AAC (*M* = 25.5 times/baseline session) but also gave directives instead of using declarative statements, inconsistently responded to Leander’s communication attempts, and did not use play strategies (e.g., setting up play routines, imitating play actions, modeling and expanding play). Rachel continued modeling AAC at a similar rate after the initial training (*M* = 26.7 times/session) and after N-AAC instruction (*M* = 28.7 times/session) but started modeling AAC more in matched turns only after N-AAC instruction. She achieved 70% fidelity immediately after N-AAC training and practice-based coaching, but she did not maintain fidelity until after three additional coaching sessions. Fidelity averaged 74.7% during the full intervention condition, with the greatest growth in declarative statements and use of play strategies (i.e., imitating play, modeling play, using nonverbal engagement strategies, expanding play). She continued to have more difficulty with limiting demands, responding to communication attempts, matching turns, and using expansions; however, these still improved meaningfully from baseline.

### 3.4. Megan (Early Childhood Assistant Teacher)

Baseline fidelity for Megan averaged 30.6%, with stronger Engagement than Language strategy use. She showed strengths in some of the Prepare strategies (physical space, toys) and letting Hallie take the lead. She had the AAC device within reach most of the time, but she rarely modeled it (*M* = 2.7 times/baseline session). She also frequently gave directives or demands, inconsistently responded to Hallie’s communication attempts, and did not use play strategies (e.g., setting up play routines, imitating Hallie’s play actions, modeling and expanding play). Megan modeled AAC more after the initial training (*M* = 6.0 times/session), including in matched turns, but this increased more after N-AAC instruction (*M* = 12 times/session). She achieved 70% fidelity in her second session after the N-AAC training sessions and practice-based coaching sessions, but she did not maintain fidelity until after three additional coaching sessions. Fidelity averaged 74.6% during the full intervention condition, during which she showed strong growth in Engagement strategies (sit at the child’s level, imitate play actions, model play, expand play) and Language strategies (declarative statements, AAC teaching words, pairing gestures with language models). She continued to have more difficulty with responding to Hallie’s communication, using nonverbal engagement strategies, matching turns, modeling AAC within matched turns, and using expansions; however, these still improved meaningfully from baseline.

### 3.5. RQ2: Impact on Students’ Communication and Language Use with Educators

**Figure 6:**
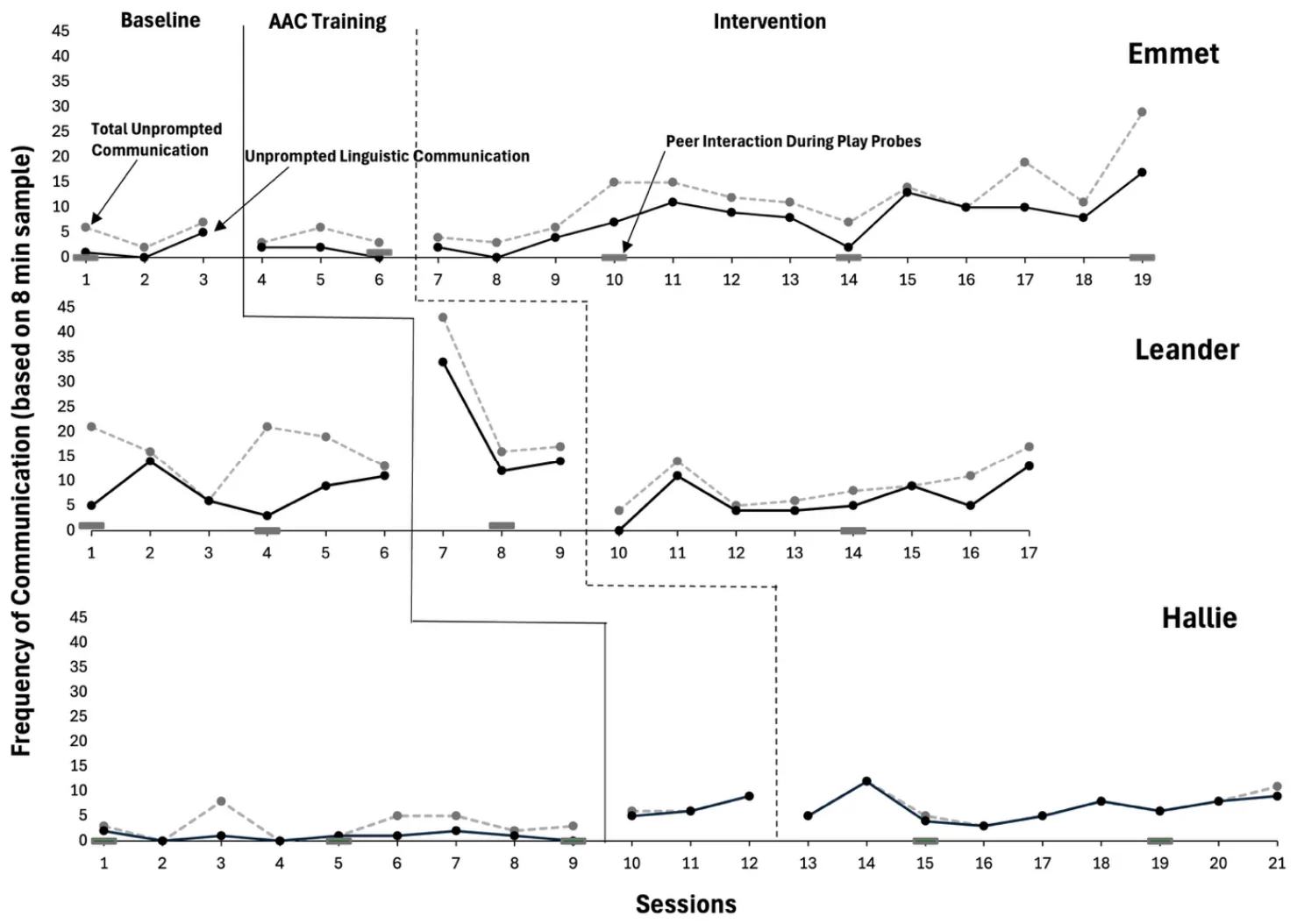
Secondary Single-Case Graph Showing Descriptive Findings about Focus Student Total Unprompted Intentional Communication and Unprompted Linguistic Communication Across Study Conditions, including in Peer Play Probes.

**Figure 7:**
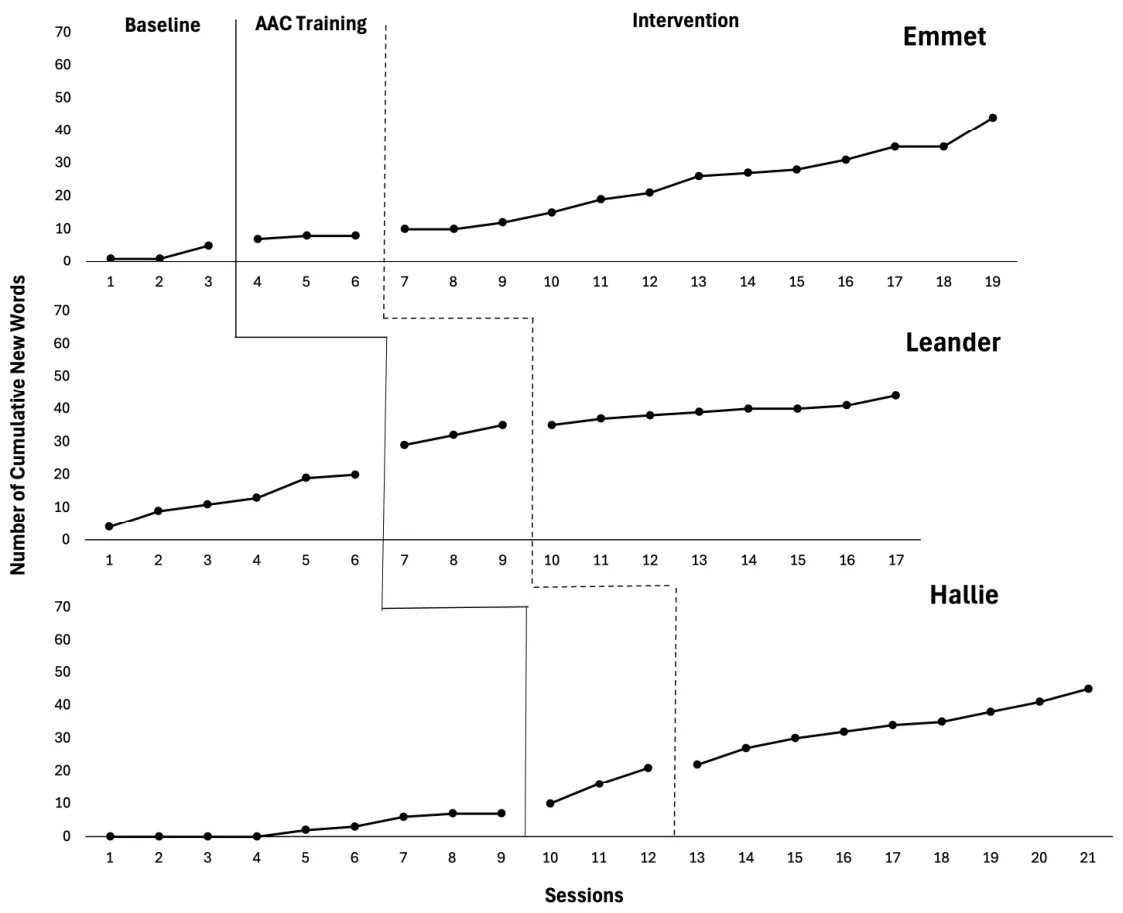
Graph of the Cumulative Number of New Words (Speech and AAC) for Focus Students across Study Conditions.

Figure 6 displays focus students’ total unprompted intentional communication and unprompted linguistic communication during educator–child play sessions. Figure 7 displays cumulative new words. Data should be interpreted descriptively, as the primary DV was educator implementation, not student outcomes. An accelerating baseline trend is expected for cumulative new words, as each session’s novel words are added to the previous count. Therefore, Figure 7 should be interpreted by visually comparing the slopes for intervention and baseline; a steeper intervention slope indicates accelerated vocabulary growth.

### 3.6. Emmet’s Communication and Language Use

Emmet’s communication was low and stable in baseline, with increasing trends during intervention for both total and linguistic communication (Figure 6). Focusing on linguistic communication, there was no change between baseline and the initial AAC training condition (*M* = 2, range 0–5 across both conditions). This increased to an average of eight linguistic communication acts during the full intervention (range, 0–17). Cumulative new words (Figure 7) showed minimal growth in baseline and the initial AAC training condition (*M* = adding 1.3 novel words/session), with steeper growth during the full intervention (*M* = 2.8 novel words/session). All unprompted words were produced with speech (100%); no AAC words were produced.

Beyond the increase in communication, there were important differences in communicative function across conditions. Behavior regulation (BR) remained stable (*M* = 2 each, range 0–5 across conditions), but joint attention (JA), spontaneous linguistic responses (RES-S), and imitated responses (RES-I) all increased (specifically during the full N-AAC intervention, not after the initial AAC training). JA averaged < 1 in baseline/initial AAC training (range, 0–2) and 2 during the full intervention (range, 0–4). RES-S averaged < 1 in baseline/initial AAC training (range, 0–2) and 2 during the full intervention (range, 0–7). RES-I averaged 1 in baseline/initial AAC training (range, 0–2) and 4 during the full intervention (range, 0–11). Despite producing only spoken words, Emmet imitated words more frequently when Abby modeled with speech and AAC versus speech alone.

### 3.7. Leander’s Communication and Language Use

Leander’s frequency of intentional communication was variable during baseline, with high amounts of non-discriminatory AAC use that is included in total communication but not linguistic communication (Figure 6). Level and variability increased after initial AAC training, particularly due to an outlying session (Session 7), but then communication frequency returned to a similar-to-baseline level (and variability) during the full intervention. Focusing on linguistic communication, average frequency was 8 during baseline (range, 3–20), 20 during the initial AAC training (range, 12–34), and 6 during the full intervention (range, 0–13). Cumulative new words (Figure 7) showed steady growth across all conditions with no slope change during intervention (*M* = 2.6 novel words/session). All unprompted words were with AAC (100%); Leander did not use any spoken words.

Three key differences emerged in communication form and function across conditions. First, Leander often demonstrated non-discriminatory AAC use during baseline and the initial AAC training condition (*M* = 4 instances across both conditions; range, 0–16), always when he was attempting to request an object; this slowed and then eventually stopped during the full intervention condition, demonstrating growth in his skills with selecting an appropriate symbol for his intended message. Second, the outlying session with the highest communication frequency (Session 7) resulted from requesting because Rachel frequently held back colored tokens that could be dropped into a toy pig’s mouth so that Leander would use his AAC device to request them. Average frequency of BR was 5 in other sessions (range, 0–12), but BR frequency was 21 in Session 7. Third, there were no JA acts in baseline and the initial AAC training condition, but JA emerged during the full intervention (*M* = 1 instance, range 0–6). JA comprised about 10% of Leander’s unprompted communication during the N-AAC condition.

### 3.8. Hallie’s Communication and Language Use

Hallie’s baseline communication was near zero, increasing after Megan’s initial AAC training and during the full intervention. Trends were similar for total and linguistic communication (Figure 6). For linguistic communication, Hallie averaged < 1 linguistic communication act during baseline (range, 0–2), which increased to 7 during the initial AAC training condition (range, 5–9), and 7 during the full intervention (range, 3–12). Cumulative new words (Figure 7) showed slow growth in baseline (*M* = 0.8 novel words/session), with a steeper slope in the initial AAC and full intervention conditions (*M* = 3.2 novel words/session). Hallie used both spoken and AAC words, often producing the same word in both modalities. Approximately one-third of her novel words were AAC produced (33.3%).

Hallie’s increase in communication was driven by increases in joint attention (JA), spontaneous linguistic responses (RES-S), and imitated responses (RES-I); the frequency of behavior regulation (BR) remained consistent across conditions. Specifically, JA averaged < 1 in baseline (range, 0–3), increasing to an average of 2 during both intervention conditions (range, 0–5). RES-S and RES-I both averaged < 1 in baseline (range, 0–2). During both intervention conditions, RES-S increased to 3 (range, 0–7) and RES-I increased to 2 (range, 0–4).

### 3.9. RQ3: Generalization to Play-Based Interactions with Peers Without Disabilities

Figure 6 displays communicative interactions between focus students and peers during play probes. The frequency of both variables was summed for graphing, due to near-zero levels. Frequency of both (a) focus student communication to peers and (b) peer communication to the focus student remained at near-zero levels in probes across all study conditions, even though all students remained in proximity to one another and peers interacted communicatively with one another. Emmet and his peers had three total communicative interactions across the five sessions. Two communicative interactions were initiations from Emmet to a peer (both were nonword vocalizations and/or gestures) (Play Probe #2 and #4). One interaction involved a peer asking Emmet for a toy (Play Probe #2). Leander and his peers had a total of two communicative interactions (across the four sessions); one involved a peer verbally offering Leander a toy (Play Probe #1), and one involved an initiation from Leander who used his AAC device to comment [COW] (Play Probe #3). The comment was not responded to by either peer. Hallie and her peers did not ever communicate with one another (across the five sessions).

### 3.10. RQ4: Social Validity

#### 3.10.1. Educator Views on the Goals, Procedures, and Outcomes

All educators reported that they valued the goals of the intervention. Related to the N-AAC strategies themselves, Abby, Rachel, and Megan rated all the strategies as either *useful* (4) or *very useful* (5) on the five-point scale. Educators also rated themselves as being comfortable or very comfortable with the strategies at the end of this study, averaging 4.8 (Abby), 4.7 (Rachel), and 4.3 (Megan) on the five-point scale. They specifically valued having new ways to support children’s engagement. Abby said she appreciated “how applicable the strategies are to other students.” Rachel explained that before the study she “usually just set up situations that would elicit a request” because she found it difficult to engage with Leander’s unique play style. She elaborated:

This project showed me fun and motivating ways to help [Leander] communicate about the things that are most interesting and important to him. It helped so much to have a collection of strategies available to me to help facilitate more communication and engagement. I was less likely to draw a blank and could always go back to copy-talk to give me a moment to think of some other ideas to expand play.

None of the educators identified disliked aspects of N-AAC, but the parts that were most difficult were: (a) engaging with students in their play, particularly when students played in unconventional ways (Rachel and Abby) and (b) modeling AAC, particularly in matched turns (Megan).

In their interviews, educators also praised the training and coaching. Rachel and Megan described the coach as “responsive”, “flexible”, and “encouraging.” Abby shared that she liked that she could “brainstorm multiple ideas” when challenges arose. Educators liked the training content and suggested: (a) finding ways to improve the memorability of strategies and (b) increasing the amount of in-person, practice-based coaching. All educators felt others would want to participate in and would benefit from professional development.

Regarding outcomes, all educators reported observing meaningful growth in communication. The pre-service teacher in Hallie’s classroom shared: “[Student] has had a language boom over the last few weeks. This intervention is definitely a big part of her success!” In addition to communication growth, Rachel emphasized the “progress with engagement” Leander made. All the educators planned to continue using N-AAC strategies. Rachel concluded, “This taught me how to provide services in the future for all my students with AAC devices!”

#### 3.10.2. Educator Ratings on Baseline and Intervention Sessions

Supplementary Table S1 displays each educators’ ratings about the quality of baseline and intervention clips; ratings were consistently higher in intervention than baseline. Average ratings for student-related items were: (a) student engagement with the adult (*M* = 2.3 in baseline and 4.2 in intervention), (b) student engagement with toys (*M* = 3.7 in baseline and 4.5 in intervention), and (c) student communication (*M* = 2.3 in baseline and 4.0 in intervention). Ratings for educator-related items were: (a) support for children’s engagement (*M* = 1.9 in baseline and 3.9 in intervention), (b) responsiveness to the child (*M* = 2.8 in baseline and 4.7 in intervention), and (c) support for communication (*M* = 2.1 in baseline and 4.1 in intervention).

## 4. Discussion

AAC-supported NDBIs offer promise for promoting language and communication development for children with autism who are minimally speaking (Crank et al., 2021; Pope et al., 2025). We evaluated whether a professional development package consisting of training and coaching could equip three educators to implement an AAC-supported NDBI. We also descriptively examined changes in students’ communication and language use. The professional development package was effective at teaching educators to implement the NDBI, called N-AAC, with fidelity. Students showed meaningful communication changes through descriptive increases in total and linguistic communication, vocabulary growth with speech and/or AAC, stronger discrimination of AAC symbols, and increases in joint attention initiations and social responses to adult utterances. However, as anticipated, these gains did not generalize to peer interactions. This study offers meaningful implications on supporting communication development for minimally speaking autistic students through educator professional development.

First, all three educators had vast differences in professional background and prior AAC training but successfully learned to implement N-AAC with fidelity, at least within the immediate research context. Rachel was a seasoned SLP with 12 years of experience, Abby was a pre-service special educator pursuing her master’s degree, and Megan was an early childhood assistant teacher in her first year in a classroom role. The educators’ varying background experiences and knowledge likely impacted many aspects of their experiences, including the range of how they implemented N-AAC strategies during baseline. Baseline implementation ranged from approximately 30% (Megan) to 50% (Abby), consistent with prior research indicating educators and other service providers often lack adequate pre-service and in-service training to implement NDBI and AAC-related strategies with fidelity (Brady et al., 2010; D’Agostino & Frost, 2025). However, all three educators implemented N-AAC with fidelity after receiving the full professional development package. The coaching model incorporated collaborative goal setting and action planning, real-time feedback and modeling, and regular reflection using fidelity data to guide practice adjustments. Thus, this study not only adds to the literature highlighting the effectiveness of collaborative coaching for educators’ use of evidence-based practices (Snyder et al., 2015), but it also demonstrates that helping educators implement AAC within an NDBI approach requires more than just basic training. At the same time, it raises the need for future research focused on understanding whether educators can maintain fidelity of implementation on their own, or whether additional support might be required for long-term success.

Second, educators brought unique strengths to N-AAC implementation, but their shared challenges illuminate needs for professional development—starting with AAC modeling itself. During baseline, Rachel modeled AAC frequently but not necessarily in back-and-forth matched turns contingent to Leander’s play and communication. Abby and Megan used AAC far less often. Although aided language modeling emerged in the AAC research literature in the late 1980s and 1990s (e.g., Goossens’, 1989; M. A. Romski & Sevcik, 1988), its mainstream adoption has only started to occur decades later, likely ushered by tablet and touchscreen accessibility (Sennott et al., 2016). Use of aided language modeling as the foundation for AAC intervention represents a significant departure from earlier approaches, which often focused on decontextualized instruction for narrow pragmatic functions, particularly requesting (Spencer et al., 2025). Importantly, increased use of aided language modeling in practice (such as by Rachel in baseline) is a crucial advance for the field. Now, this practice should be further built upon through interventions that equip educators and service providers to support children’s engagement as a crucial part of their learning. AAC-related professional development must teach educators not simply to model AAC but to model AAC in a way that supports back-and-forth interaction and to provide language models during times of joint engagement by responding contingently to children’s communication and play.

Other challenging aspects of N-AAC implementation also pinpoint strategies to focus on within professional development. At baseline, no educator consistently (a) used play strategies such as imitating, modeling, and expanding play or (b) responded to focus student communication. First, play strategies are crucial because they scaffold joint engagement to create learning opportunities (Bottema-Beutel et al., 2014). One of the most meaningful educator changes that resulted from the N-AAC instruction was educators developing and using skills to imitate and expand children’s play. Rachel’s social validity feedback exemplified this when she described how helpful the “copy-talk” strategy was (i.e., copy = imitate play actions, talk = model language with speech or AAC about the play action through a matched turn). This simple strategy transformed her ability to create powerful learning opportunities for Leander. Second, recognizing and responding to children’s communication (including prelinguistic communication and PCBs) reinforces communication attempts and creates opportunities to assign meaning and expand language (McDaniel et al., 2022). Although educators’ use of communication responses, matched turns, and language expansions did improve, these remained more challenging than other N-AAC strategies. These strategies require educators to interpret children’s subtle communication, resist their own impulses to be directive, and generate responsive language in real time. Therefore, the findings underscore how educators need ample practice and support to develop and maintain these cognitively demanding skills.

Third, focus student communication and language use varied, but students showed unique patterns of descriptive growth that included increased communication frequency (Emmet and Hallie), new use of words with speech and/or AAC (Emmet and Hallie), and changes in communicative function that included increases in joint attention and responses to adults’ initiations (Emmet, Leander, and Hallie). Leander was already communicating at higher rates in play sessions than the other two students, though primarily to request and brought on by Rachel’s use of time delay. His communication frequency did not increase during N-AAC implementation, but he did expand his use of communicative functions, particularly the emergence of joint attention initiations. This shift is noteworthy because joint attention is foundational for social interaction but substantially more challenging for children with autism to develop than requesting (Spencer et al., 2025).

Somewhat differently, Emmet and Hallie had very low baseline communication that increased meaningfully during N-AAC implementation, namely through increases in social responses (both spontaneous and imitated), along with joint attention initiations. Hallie’s communication frequency began increasing during the initial AAC phase, which could have been related to Megan beginning to use aided language modeling after this initial training. Emmet’s communication frequency did not increase until the full N-AAC condition. Hallie used both spoken and AAC words, whereas Emmet relied exclusively on speech. Several factors could explain why Emmet did not use his AAC device expressively, most notably that he was introduced to AAC during this study, rather than having used it before. Emmet may have needed more time to learn to navigate the AAC system (e.g., vocabulary organization, developing motor plans for specific words) to use it to communicate. At the same time, however, it is interesting that Emmet imitated Abby’s language models more frequently when she combined AAC and speech, compared to speech alone. A plausible explanation is that AAC modeling might have enhanced the salience of the language input for him. Although this descriptive finding should be interpreted cautiously, it aligns with broader evidence that AAC has important roles as a tool for language learning and comprehension, not just for expression (Biggs et al., 2018; Wood et al., 1998).

Across all three students, educators recognized these gains as meaningful. However, despite strong outcomes in the context of adult–child interactions, no generalization to peer interactions occurred. Focus students almost never interacted with peers, and peers almost never interacted with them. This finding was anticipated and should be interpreted cautiously. Nonetheless, the finding still carries empirical and practical significance. Although generalization to new communication partners is a known challenge for children with autism (Hampton et al., 2021), this specific hypothesis had not been systematically tested within AAC-supported NDBIs. Peer interaction and engagement are crucial for the development and wellbeing of all children (Biggs & Rossi, 2022). Therefore, educators likely need training and support not only to implement AAC-supported NDBIs with fidelity but also to support peers as responsive communication and inclusive play partners.

### Limitations and Directions for Future Research

Several limitations warrant consideration and inform needs for future research. First, study coders were not blind to study conditions due to practical constraints for the research team, a common challenge in single-case design studies. Second, we could not conduct planned maintenance probes because recruitment and initial planning steps (e.g., child assessments, AAC device trials, scheduling, recruiting of peers) took longer than anticipated. Future research must examine whether educators sustain N-AAC implementation over time, as maintenance is critical for meaningful and durable intervention effects. Third, our focus on educator implementation provides needed insights into whether educators can learn to implement N-AAC with fidelity, but it restricts the claims we can make regarding the intervention’s impact on student outcomes. Future research is needed using designs that allow for more rigorous measurement of student impact, particularly students’ communication skills beyond the immediate intervention context (Yoder et al., 2018). Fourth, our IOA was generally strong, but IOA was low (60%) for focus student communicative function in one of the individual sessions. Low IOA in this session resulted from limited opportunities to score function (i.e., low frequency of focus student communication makes each disagreement carry greater weight in the percentage). Fifth, we collected social validity perspectives from educators, including those who implemented N-AAC and others who did not. However, we did not collect social validity reports directly from families or students with disabilities themselves. Future research is particularly needed to determine accessible ways to allow minimally speaking autistic students to share views on the acceptability of AAC interventions like this one. Last, the use of a single-case design necessitated staggered introduction of the intervention, and so we conducted 1:1 training sessions with educators. However, such a training model does not fully account for the complex realities of school environments, including educator caseload demands, financial constraints, and other resource limitations. Future work is needed to examine the potential for larger-scale implementation of N-AAC at the school or district level, which will require close partnership with local educational agencies to ensure that N-AAC strategies and professional development modes are feasible, sustainable, and aligned with the everyday contexts in which educators work. This includes a need for future research focused on implementing N-AAC in ordinary classroom routines and inclusive activities with peers.

## 5. Conclusions

Communication is a fundamental right for all children, yet many minimally speaking autistic students lack adequate support for their language development in school settings. This study evaluated a professional development package consisting of training and coaching to teach three educators with diverse professional backgrounds to implement N-AAC, a play-based AAC-supported NDBI with fidelity. All three educators successfully implemented N-AAC following professional development, and their students descriptively demonstrated individualized but meaningful changes in their communication, including related to frequency, vocabulary growth, and increases in joint attention and social responses. However, as anticipated, these gains did not generalize to peer interactions. Together, these findings highlight the importance of training and coaching for educators on engagement and language-related strategies for AAC intervention, as well as supporting peers as responsive communication partners for minimally speaking autistic students.

## Data Availability

The data presented in this study are available on request from the corresponding author.

## Note

1 The words used to describe people are powerful. We alternate between person-first (student with autism) and identity-first language (autistic students) to acknowledge there are different preferences. We encourage readers to explore resources about the words that self-advocates prefer, including the words self-advocates with communication-related disabilities use to describe themselves. One such research has been published by CommunicationFIRST and can be accessed at https://communicationfirst.org/wp-content/uploads/2023/07/C1st-The-Words-We-Use-Style-Guide-v1-July-2023.pdf (accessed on 1 June 2026).

## Supplementary Materials

The following supporting information can be downloaded at https://www.mdpi.com/article/10.3390/bs16081275/s1, Table S1: Social validity ratings from school staff members related to the quality of student-adult interactions during baseline and intervention play sessions.
