# Supplementary material for: Play-Based Naturalistic Augmentative and Alternative Communication (AAC) Intervention: Educator Fidelity and Effects on Communication and Peer Interactions
Source: Behav Sci (Basel). 2026 Jul 27;16(8):1275. doi: 10.3390/bs16081275 (PMC13509394; doi:10.3390/bs16081275)
Supplement: Supplementary file 1 [file behavsci-16-01275-s001.zip › behavsci-4410340-supplementary.pdf]

Table S1. Social validity ratings from school staff members related to the quality of student-adult interactions during baseline and intervention play sessions

	Baseline						Intervention					
	Emmet		Leander	Hallie			Emmet		Leander	Hallie		
	Abby	Other	Rachel	Megan	Other	*M*	Abby	Other	Rachel	Megan	Other	*M*
Student engagement with adult	1/1	3/4	2/2	2/3	2/3	**2.3**	4/3	5/5	5/4	4/5	4/3	**4.2**
Student engagement with toys	4/3	3/4	2/2	5/5	5/4	**3.7**	5/5	5/5	5/3	5/5	4/3	**4.5**
Student communication	2/1	3/3	2/1	4/2	2/3	**2.3**	4/3	5/5	4/3	4/5	4/3	**4.0**
Adult support for engagement	1/1	3/3	2/3	1/1	2/2	**1.9**	4/3	5/5	4/3	3/4	5/3	**3.9**
Adult responsiveness	4/2	3/3	3/2	2/3	3/3	**2.8**	5/4	5/5	5/5	4/5	5/4	**4.7**
Adult support for communication	1/1	3/3	2/3	1/2	3/2	**2.1**	4/3	5/5	4/4	3/4	5/4	**4.1**

Note. School staff provided ratings on two randomly selected video clips from baseline sessions, and two other randomly selected clips from intervention sessions. For most items, data reflect the school staff member's rating on the topic using a 5-point scale: 1 = *very low*, 2 = *low*, 3 = *moderate*, 4 = *strong*, 5 = *very strong*; numbers displayed are separated by a / for the two different clips (i.e., rating for clip #1/rating for clip #2).

M = mean across all school staff ratings for the two video clips from that study condition
